# An overview and prediction of 100 mm performance over 40 years during the Athletics World Championships

**DOI:** 10.5114/biolsport.2025.146783

**Published:** 2025-04-01

**Authors:** Krzysztof Kotuła, Irineu Loturco, Aleksander Matusiñski, Adam Zając, Adam Maszczyk

**Affiliations:** 1 Institute of Sport Sciences, The Jerzy Kukuczka Academy of Physical Education, Katowice, Poland; 2 Nucleus of High Performance in Sport, Sao Paulo, Sao Paulo, Brazil; 3 Department of Human Movement Sciences, Federal University of Sao Paulo, Sao Paulo, Brazil; 4 University of South Wales, Wales, United Kingdom

**Keywords:** Athletic performance, Track and field, Speed, Elite athletes, Sprint velocity

## Abstract

The primary aim of this study was to determine whether the significant improvements in 100 m-meter sprint times over the past 40 years are the result of an overall enhancement in performance among all elite sprinters, or are mainly driven by the sporadic emergence of exceptionally talented individuals. Additionally, we compared the average age of the semifinalists with that of the champion. To explore this broader research question, we compared the average performance times and ages of World Championship semifinalists with those of the champions over successive 2-year intervals. This approach allowed us to ascertain whether there has been a consistent improvement in average performances among elite sprinters or whether progress is mainly due to extraordinary athletes who occasionally set new records. By analyzing these patterns, we aimed to understand the underlying factors contributing to advancements in sprint performance and to assess whether these improvements are widespread or concentrated among a few exceptional individuals. Finally, we analyzed the relationship between age and 100 m-meter performance and predicted the results of semifinalists and winners at the Athletics World Championships in 2025 and 2027, for both men and women. The results obtained suggest that progress in sprinting, in both sexes, depends on the emergence of exceptionally talented individuals who set new world records or achieve world-leading times during the main competitions of the season. These exceptionally talented athletes have improved the winning times in the main 100 m-meter competitions, while the average times of semifinalists at the Athletics World Championships have remained relatively constant, ranging between 10.40 and 10.50 seconds over the analyzed 40-year period.

## INTRODUCTION

The 100 m m sprint results have been marked by meaningful progress over the years, driven by advancements in training methods, sports selection, nutrition, equipment and recovery procedures [1–3]. In the early years of modern athletics, sprint times were relatively slow compared to modern standards. In the late 19^th^ century and early 20^th^ century, athletes trained occasionally on an irregular basis and mostly competed on cinder tracks without specialized footwear. In the second part of the 20^th^ century, sprint times improved dramatically due to more sophisticated training methods, the introduction of synthetic tracks, lighter and more advanced spikes, and the implementation of starting blocks. Timing became more consistent and precise with the adoption of electronic measurements and photo finishes. One factor that influenced the acceleration phase of sprinting was undoubtedly the selection of extraordinary talents from around the world, resulting in the greater popularity of track and field, especially the 100 m m sprint events [2]. Olympic and World Champions such as Carl Lewis, Usain Bolt, Florence Griffith-Joyner and Shelly Ann Frazier-Price became role models, and for the first time, financial benefits emerged from the track. The 21^st^ century has witnessed numerous world records, with Usain Bolt demonstrating absolute dominance in the 100 m and 200 m sprint events. Analyses of world record performances have been conducted since the IAAF recognized the first world record established by the 1912 Olympic winner Donald Lippincott with a time of 10.6 s. More advanced analysis and predictions have been carried out since 1968 following the introduction of electronic timing. However, comparisons and predictions of 100 m m sprint performances are quite difficult due to numerous factors affecting sprint results. These factors include wind, altitude, temperature, humidity, lane draw, track surface, spike technology, clothing, as well as training, nutrition, and recovery [4–7]. These technological innovations implemented into training and competition make comparisons difficult. Yet, despite these advances, records set by Bolt and Griffith-Joyner stand out, and it seems that they will not be broken in the near future. Thus, the scientific and coaching debate continues: are sprinters born or made? How do selection and talent affect performances, and what are the main limitations and the window of development for sprinting speed in the human body?

Some authors have indicated that geographical variables influence the development of athletic performance and, in some cases, sprinting excellence [8–11]. Considering Olympic and World Champions, the majority is of West African origin, with most winners coming from the USA or Jamaica. However, the sprinting phenomenon extends beyond simple geography; it involves a complexity of socio-economic, cultural, infrastructural, and other factors that affect both access and opportunities to participate in sports, profoundly influencing the processes of talent identification and development [11–13]. Accordingly, the evolution of the 100 m m sprint results reflects the pursuit of athletes of both sexes in the quest to maximize human performance in the realm of sprint speed and athleticism. The 100 m m sprint is perhaps the most prestigious event during the Olympic Games, and Olympic champions are regarded as sporting legends. Records continue to be broken, and new milestones are reached as the legacy of sprinting continues to inspire generations of track and field athletes worldwide.

Maximum sprint speed, which is a product of step length and step frequency, depends on various factors. These factors are related to morphological and physiological characteristics, energetic mechanisms, age, gender, motor abilities, inter- and intra-muscular coordination, and optimal sprinting technique [14–18]. Sprint speed is one of the most important physical abilities, which defines the success of athletes in many sport disciplines [19–20]. From the genetic point of view (hereditary), speed can be classified into primary phylogenetic human movements. In specific sports situations, speed is manifested in the form of the three-segment model [21–23]. The model consists of speed, strength, and coordination. The emphasis on individual segments of this model depends on the specificities of a given sport discipline. As previously mentioned, sprinting speed is a product of both the frequency and the length of stride [15, 22, 24, 25]. These variables are mutually dependent; they are also linked to the processes of central regulation of movement, morphological characteristics, motor abilities, and exercise metabolism [26, 27]. The relationship between the frequency and the length of a stride is individually defined and automated. Changing one variable results in changes in the second. When the length of a stride is increased, the frequency decreases, and vice versa. With increased speed, both variables increase [25, 26, 27].

Considering the above, the main objective of the current study was to compare the average results of world championship semifinalists against the results of champions over successive 2-year time intervals. Additionally, we compare the average age of the semifinalists with the age of the champion. The World Championships were chosen instead of the Olympic Games because of the time frame as they take place every two, and not four years. Such an approach gave us more time points for analysis. We considered only results with electronic timing which was introduced in the second half of the XX century. We attempted to determine whether the average 100 m m results have improved significantly over the past 40 years or whether record-breaking performances are the domain of exceptional athletes that appear occasionally. Additionally, we analyzed the relationship between age and 100 m m performance and predicted the results of sprinter semifinalists and winners for the 2025 and 2027 Athletics World Championships, for both men and women.

## MATERIALS AND METHODS

The research material consisted of databases of individual men’s and female’s competitions obtained from the International Association of Athletics Federations (IAAF) website. Since the data provided on the website only cover the period from 1990 onwards, earlier ranking lists were obtained electronically and sent in spreadsheet form. An additional database was obtained directly from the editorial office of the specialized track and field athletics magazine “Track and Field” in the United States. To avoid mistakes or discrepancies, both databases were compared and verified against each other. For complete certainty, the prepared material was re-verified by comparison with data provided by “The-sports.org Company”. This material was electronically transmitted in the form of a database report (records) and a spreadsheet file. Most of the data available for comparisons are on the Canadian website: http://www.the-sports.org/.

### Considered variables

Since athletics now encompasses a relatively large number of events, this study focused on analyzing the results of men’s and women’s 100 m m sprints. Many researchers have analyzed the dynamics of sports results considering the development of World or European records over time and separately analyzing the results of particular seasons and Olympic Games [26, 27]. Due to the variety of random and immeasurable factors affecting record performances, it has been unequivocally demonstrated that more accurate results of developmental trends in particular disciplines and events can be obtained by examining a series of leading performances based on the arithmetic mean values of the best achievements worldwide in successive, equally spaced time intervals. This approach also allows, among other things, the determination of how far a record in a given event deviates from the average achievements of other athletes, thus showing by how much an individual achievement – through a fortuitous combination of favorable factors – exceeds the overall level of development in that event.

Another issue to address was the multitude of different athletics competitions held annually in various parts of the world and the appropriate time frames. Therefore, based on the research assumptions, we decided to build a database using the results of the men’s and female’s 100 m m sprint performances at the Athletics World Championships from the inaugural edition in 1983 in Helsinki to the latest one in Budapest in 2023. Additionally, to maintain temporal consistency and establish optimal trend lines for predicting results, it was decided to include the World Cup results from 1985 and 1989 in the analysis. In those years, unlike subsequent ones, the Athletics World Championships were not organized. Following this, global events occurred every two years, except during the period of the COVID-19 pandemic.

Of course, it would be more advantageous if the averaged results came from the same competitions or events for the top eight athletes. In that case, all athletes would have experienced the same external conditions, making the results more reliable. However, this requires selecting certain periods (events) for analysis while disregarding others. The downside of this solution from an analytical perspective is the inclusion of a time jump without being able to demonstrate delays. In addition, the moving average and indexing could potentially underestimate certain aspects, hence it is necessary to check the degree of fit of the trend function to the empirical data each time before forecasting, using a convergence coefficient.

In the presented study, the average value of the 16 best results from the semi-finals and the results of the final races without their best results (using 16 semi-final results to standardize the data, as since 1999, 24 athletes have competed) was adopted for analysis (Table 1). A 2-year trend was chosen due to the 2-year interval in the arrangement of tables and records in the databases. Missing data for individual years for certain races (due to disqualification, injuries, etc.) were estimated using statistical inference methods, specifically estimation of unknown parameter distribution values.

**TABLE 1:** Characteristics of the semifinalists average results, standard deviation (SD) and the standard error (SE).

Variable	Men	SD	SE
1983			
Mean Women	11,444	0.030	0.017
Mean Men	10,573	0.029	0.015

1987			
Mean Women	11,346	0.020	0.011
Mean Men	10,522	0.027	0.010

1991			
Mean Women	11,241	0.022	0.010
Mean Men	10,423	0.033	0.009

1993			
Mean Women	11,153	0.023	0.012
Mean Men	10,454	0.031	0.011

1995			
Mean Women	11,159	0.030	0.010
Mean Men	10,463	0.030	0.007

1997			
Mean Women	11,233	0.015	0.004
Mean Men	10,471	0.016	0.002

1999			
Mean Women	11,024	0.024	0.005
Mean Men	10,384	0.012	0.001

2001			
Mean Women	11,285	0.026	0.009
Mean Men	10,465	0.015	0.007

2003			
Mean Women	11,147	0.010	0.008
Mean Men	10,430	0.012	0.002

2005			
Mean Women	11,133	0.017	0.0105
Mean Men	10,458	0.012	0.008

2007			
Mean Women	11,131	0.015	0.007
Mean Men	10,487	0.011	0.005

2009			
Mean Women	11,346	0.015	0.004
Mean Men	10,457	0.010	0.003

2011			
Mean Women	11,329	0.014	0.003
Mean Men	10,409	0.010	0.004

2013			
Mean Women	11,187	0.014	0.009
Mean Men	10,461	0.012	0.007

2015			
Mean Women	11,026	0.011	0.006
Mean Men	10,436	0.010	0.006

2017			
Mean Women	11,096	0.017	0.001
Mean Men	10,395	0.003	0.007

2019			
Mean Women	11,112	0.012	0.005
Mean Men	10,476	0.033	0.010

2022			
Mean Women	11,020	0.010	0.004
Mean Men	10,394	0.011	0.008

2023			
Mean Women	11,028	0.011	0.005
Mean Men	10,428	0.012	0.003

The data were obtained from publicly available sources, such as official IAAF websites or other sports databases. Since the data on sports results are public and do not contain sensitive personal information, the study did not violate the privacy of the athletes.

Only official results approved by the IAAF were considered. The results of disqualified athletes were excluded from the analysis.

### Statistical analysis

To characterize the structure of the examined variables, basic descriptive statistics were calculated in the form of measures of central tendency (i.e., mean) and measures of variability (i.e., standard deviation). Both the results and input data were presented as records in a tabular matrix.

The distributions of the tested variables were verified using the Shapiro-Wilk test for normality. The homogeneity of variances was checked with Levene’s test. In summary, all variable variances had a normal distribution with slight left or right deviations, which, however, fell within the normal range. Additionally, the significance level for Mauchly’s test was checked. Since the results were statistically insignificant, these outcomes indicated the sphericity of variances. The Shapiro-Wilk tests indicated a violation of normality for the following variables: 100 m m men – best result, 100 m m female – best result, 100 m m men – average result, and 100 m m female – average result.

In the initial stage of empirical research, a time series was used to investigate the dynamics of the phenomenon. In this approach, the levels of the variable – sprint performance – were considered as a function of time. Introducing a time unit numbering from t = 0 to t = n-1 and assigning observed levels of the studied phenomenon to these numbers yielded what is known as a realization of a stochastic process in the form of a time series. To analyze the variability of the phenomenon’s dynamics, indices based on both variables (i.e., chain) and constant bases were utilized.

The dynamics of the results were analyzed using indices based on fixed and variable time series. Using moving averages, the trend function was determined and selected as follows:


f(t)=a+b⋅t

where:

“a” represents the trend value at period 0,

“b” denotes the average periodic increment (b > 0) or decrement (b < 0) of the trend,

“t” is the time variable.

Before predicting the magnitude of the observed phenomenon for subsequent periods, the degree of fit of the trend function to the empirical data was verified. To do this, convergence coefficients were calculated and expressed by the formula:


$$\phi^{2}=\frac{\sum {\left[x_{i}+f(t)\right]}^{2}}{\sum {\left(x_{i}-\bar{x}\right)}^{2}}$$

To predict the outcome of a given competition, a time series model was applied according to the formula:


$$y\;=\;\beta_{0}+\beta_{1}x_{1i}+\beta_{2}x_{2i}+\ldots \beta_{k}x_{ki}+\varepsilon_{i}$$

for: i = 1, 2, … ,n

To verify the significance differences between results, repeated measures analysis of variance (ANOVA) was applied. In case of significant differences, further analyses were conducted using the Tukey post-hoc test. The F-statistic and significance levels were presented. The ANOVA was applied to compare the average results of semifinalists in successive World Championships, where the number of observations and their variance allow the use of this statistical method. World records were included in the analysis only as a reference point and were not directly subjected to statistical analysis using ANOVA. Correlations analyses were conducted to show dependencies between the average 100 m m times and ages of men and female semifinalists, as well as the time and age of the winners of the Athletics World Championships in respective years.

In the second stage of prediction-focused research, predictive models were constructed based on the developed data. In the case of regression models, it is assumed that relationships between variables are inherently linear. Furthermore, multiple linear regression also requires the assumption that the relationship between variables is linear. In practice, confirming the validity of such an assumption is almost impossible; however, multiple regression procedures are quite resilient to small deviations from this assumption. Nevertheless, since sports phenomena analyzed using correlograms suggested non-linear dependencies (such as a result parabola as a function of time) for the majority of tested variables, it was decided to utilize both analog and neural nonlinear models. This ensured that a type II error would not be committed during the estimation of predictive values and provided the possibility of nonlinear estimations, where we determined the nature of this relationship ourselves. For example, we can assume that the outcome variable should be a logarithmic function of independent variables, an exponential function, a moving average function, etc. [31–35].

The determination and construction of predictive value models began with time series of the periods analyzed. Moving average prediction was adopted as the forecasting method. The coefficient of variability values were monitored each time. Next, nonlinear regression models were constructed in tabular form using an Excel spreadsheet. The forecasting of nonlinear regression models was based on the power method and exponential smoothing. When we determine that unit increases in the explanatory variable corresponds to increasing or decreasing values in the dependent variable, the regression function model may take the form:


$$y=b_{0}b_{1}^{x}$$

Therefore, the regression models were determined using regression and tabular functions. The most important part of this stage was the construction and forecasting using neural models. It was based on models with radial basis functions (RBF), due to the different distribution of input data into the models. Time series forecasts using artificial neural networks were determined by selecting the network structure with the lowest validation error values from among all tested ones. For all analyses, a statistical significance level of p < 0.05 was adopted. All calculations were performed using the Statistica v.15 software (StatSoft, USA, CA).

## RESULTS

### 100 m m Men

Table 2 presents average times and ages of men semifinalists, as well as the time and age of the men’s winner of the Athletics World Championships in respective years. Table 3 indicate correlational dependencies; however, these were not very strong. Analysis of correlations among men indicates that the older the winner, the worse the result achieved. Conversely, in the case of semifinalists and other finalists, the younger the competitor, the better the result.

**TABLE 2:** Comparison of the average times and ages of men semifinalists, as well as the time and age of the men’s winner at the Athletics World Championships in respective years.

World Championships	Winner	Country (winner)	Age (winner)	Time (winner)	Average age (semi finalist)	Average time (semi finalist)
1983	Carl Lewis	USA	22	10.07	24	10.58
1985	Ben Johnson	CAN	21	10.00	23	10.56
1987	Carl Lewis	USA	26	9.93	25	10.53
1986	Linford Christie	GBR	29	10.10	25	10.49
1991	Carl Lewis	USA	30	9.86	27	10.43
1993	Linford Christie	Jamaica	33	9.87	28	10.46
1995	Donovan Bailey	Jamaica	28	9.97	25	10.47
1997	Maurice Greene	USA	23	9.86	22	10.48
1999	Maurice Greene	USA	25	9.80	25	10.39
2001	Maurice Greene	USA	27	9.82	26	10.48
2003	Kim Collins	Saint Peter Basseterre	27	10.07	25	10.44
2005	Justin Gatlin	USA	23	9.88	24	10.47
2007	Tyson Gay	USA	25	9.85	26	10.50
2009	Usain Bolt	Jamica	23	9.58	25	10.47
2011	Yohan Blake	Jamica	22	9.92	26	10.42
2013	Usain Bolt	Jamica	27	9.77	26	10.47
2015	Usain Bolt	Jamica	29	9.79	27	10.45
2017	Justin Gatlin	USA	35	9.92	28	10.40
2019	Christian Coleman	USA	23	9.76	25	10.49
2022	Fred Kerley	USA	27	9.86	24	10.40
2023	Noah Lyles	USA	26	9.83	23	10.44

**TABLE 3:** Results of the correlation analysis between the average times and ages of men’s semifinalists, as well as the times and ages of the winners at the Athletic World Championships in respective years.

Variables	Time achieved (winner)	Average time achieved (semifinalists)
Age in years (winner)	0.105	-0.389
Average age (semifinalists)	-0.064	-0.250

**FIG. 1:**
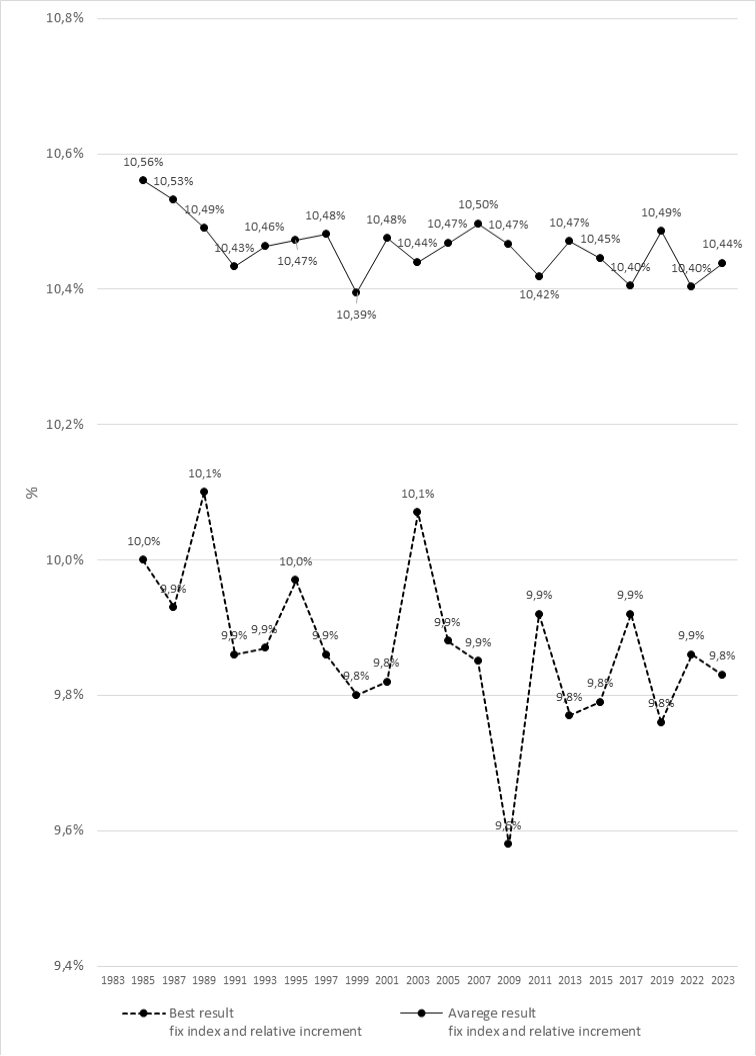
Differences between the best and average results of the men’s 100m sprint during the Athletics World Championships from 1983 - 2023 using fixed indices and relative increments.

**FIG. 2:**
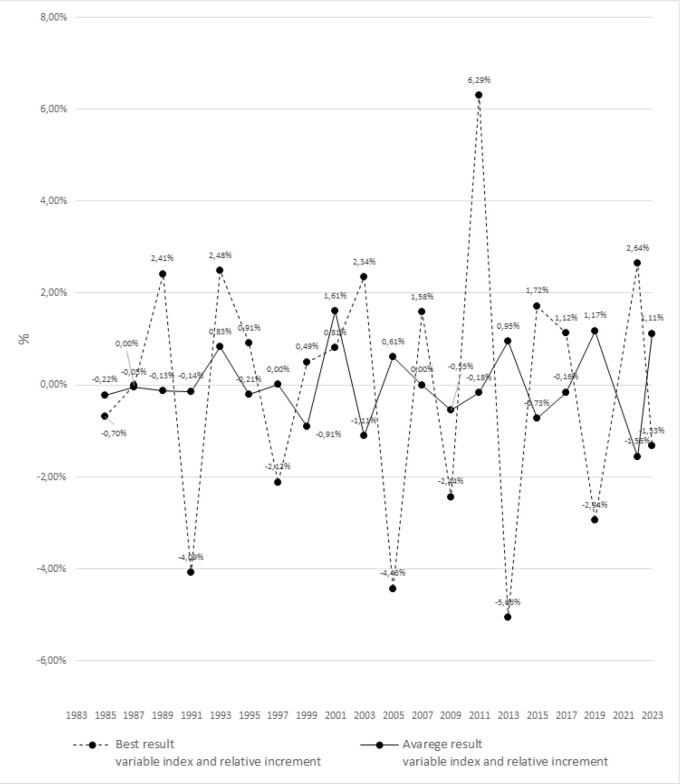
The dynamics of changes in the best and average results of the men’s 100m sprint during the Athletics World Championships from 1983 - 2023 using variable indices and relative increments.

Figures 1 and 2 present significant differences in the dynamics of changes between the best and averages results using the fixed and variable indexes in relative increments for the 100 m m men’s sprint during the Athletics World Championships between 1983 to 2023.

ANOVA with repeated measures and post-hoc tests revealed significant differences between the best and average results for all analyzed seasons. The most significant differences were detected in 1991 (p = 0.004), 2007 (p = 0.003), 2009 (p = 0.001), and 2023 (p = 0.004).

### 100 m m Female

Table 4 presents the average times and ages of female’s semifinalists, as well as the time and age of the female’s winner at the Athletics World Championships in respective years. Table 5 indicate correlational dependencies, although they were not very strong. In the case of female, both winners and other participants, younger athletes achieved better results.

**TABLE 4:** Comparison of the average times and ages of men semifinalists, as well as the time and age of the female’s winner of the Athletics World Championships in respective years.

World Championships	Winner	Country (winner)	Age (winner)	Time (winner)	Average age (semifinalists)	Average time (semifinalists)
1983	Marlies Olsner-Göhr	DDR	25	10.97	23	11.44
1985	Marlies Olsner-Göhr	DDR	27	11.10	24	11.41
1987	Silke Möller	DDR	23	10.90	22	11.35
1989	Sheila Echols	USA	22	11.18	24	11.31
1991	Katrin Krabbe	DDR	22	10.99	26	11.24
1993	Gail Devers	USA	27	10.82	26	11.15
1995	Gwen Torrence	USA	30	10.85	25	11.16
1997	Marion Jones	USA	22	10.83	23	11.23
1999	Marion Jones	USA	24	10.70	25	11.02
2001	Ekateríni Thánou	Greece	26	11.05	26	11.28
2003	Torri Edwards	USA	26	10.93	25	11.15
2005	Lauryn Williams	USA	22	10.93	24	11.13
2007	Veronica Campbell-Brown	Jamica	25	11.01	25	11.13
2009	Shelly-Ann Fraser-Pryce	Jamica	23	10.73	25	11.35
2011	Carmelita Jeter	USA	32	10.99	26	11.33
2013	Shelly-Ann Fraser-Pryce	Jamica	27	10.71	25	11.19
2015	Shelly-Ann Fraser-Pryce	Jamica	29	10.76	27	11.03
2017	Tori Bowie	USA	27	10.85	28	11.10
2019	Shelly-Ann Fraser-Pryce	Jamica	33	10.71	25	11.11
2022	Shelly-Ann Fraser-Pryce	Jamica	35	10.67	24	11.02
2023	Sha’Carri Richardson	USA	23	10.65	23	11.03

**TABLE 5:** Results of the correlation analyses between average times and age of female’s semifinalists, as well as the time and age of the winners of the Athletics World Championships in respective years.

Variables	Time of the winner	Average time of semifinalists
Age in years (winner)	-0.239	-0.273
Average age (semifinalists)	0.120	-0.267

ANOVA with repeated measures and post-hoc tests revealed significant differences between the best and average results of the 100 m m female’s sprint for all analyzed seasons, except for the year 2007 (p = 0.232). The most significant differences were noted in 1999 (p = 0.001), 2013 (p = 0.004), 2015 (p = 0.002), and 2017 (p = 0.004).

**FIG. 3:**
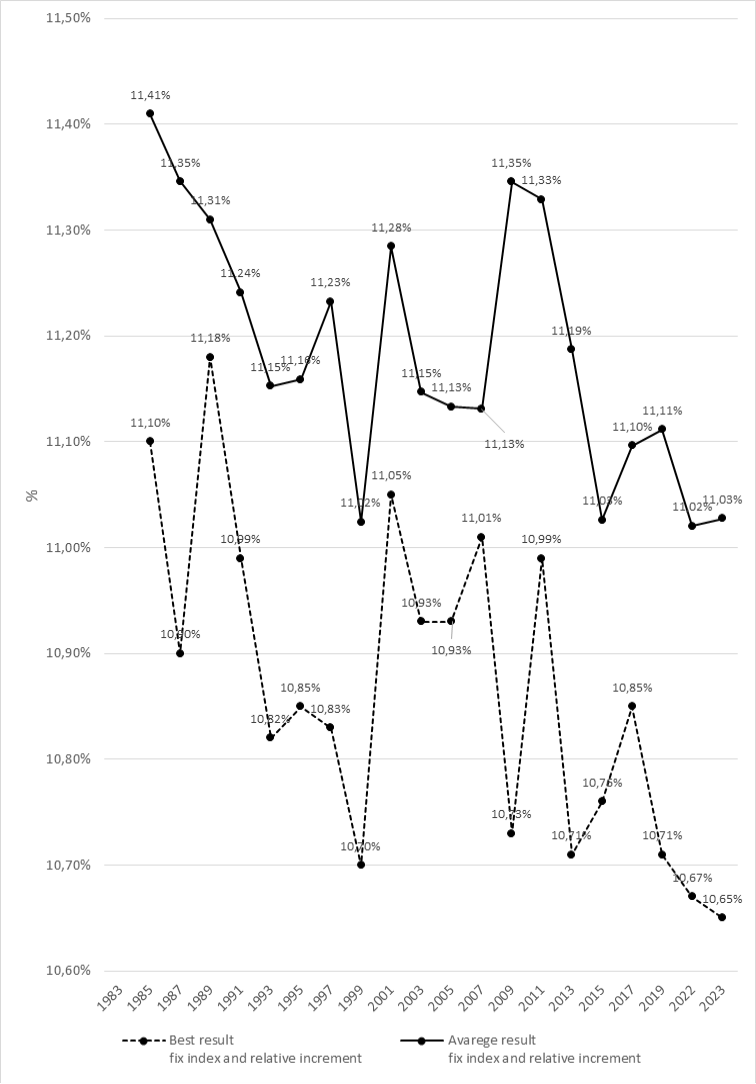
Differences between best and average results of the 100 mm female’s sprint during the Athletics World Championships from 1983–2023.

**FIG. 4:**
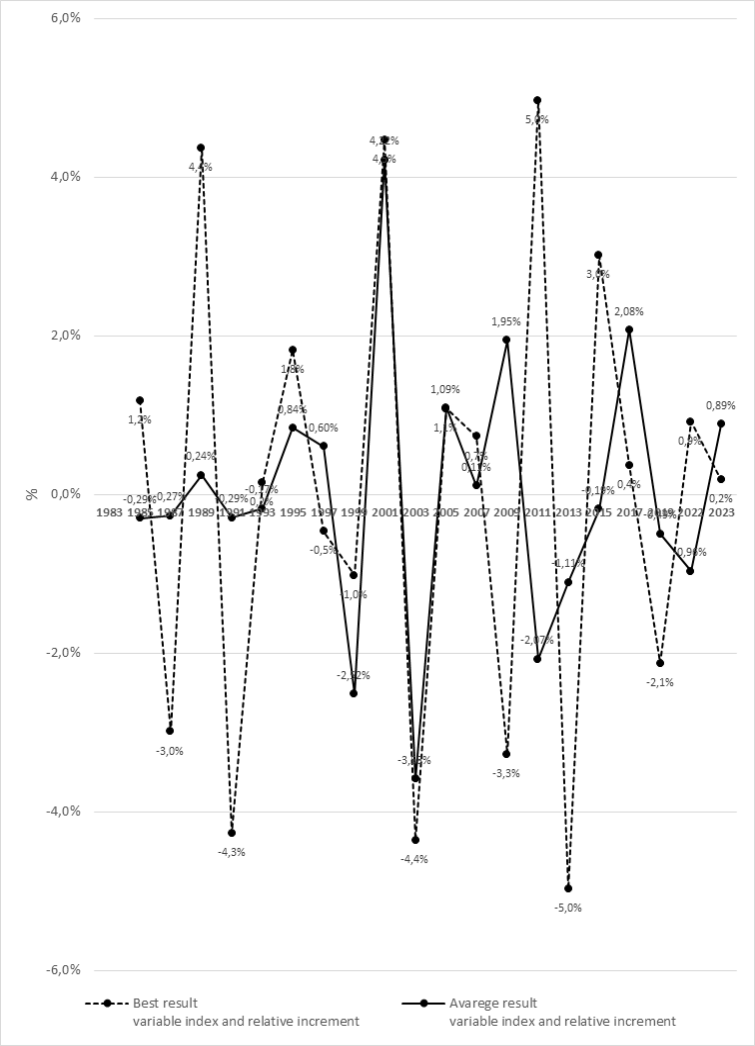
The dynamics of changes in the best and average results of the female’s 100 mm sprint during the Athletics World Championships from 1983 to 2023, using variable indices and relative increments.

Figures 3 and 4 present significant differences in the dynamics of changes between the best and averages results using the variable indices in relative increments for the female’s 100 m m sprint during the Athletics World Championships from 1983–2023.

### Prediction of 100 mm Men’s vs. 100 mm Female’s sprint times for the 2025 and 2027 Athletics World Championships

ANOVA with repeated measures and post-hoc tests revealed significant differences between the best and average results of 100 mm sprint times for female and men across all analyzed seasons during the Athletics World Championships since 1983.

First, the predictive models for the year 2023 were examined based on known historical results and the best and average results during the 2023 Athletics World Championships. Comparing the models among themselves and with the results of sprint competitions at the 2023 Athletics World Championships allows us to determine the value and accuracy of the predicting results to some extent. Table 6 presents the predictions for the year 2023 and compares them with the current best and average results of the 100 mm races for female and men during the 2023 Athletics World Championships. As seen in both cases, for female and men, the neural models with an acceptable error (± 0.01) predicted the best results achieved in 2023.

**TABLE 6:** Predictive values of the models and the average results obtained at the 2023 Athletics World Championships in the female’s and men’s 100 mm race

Competition	Gender	Predictions for place	Year	RM	TSPM	RBF 1-3-1	World Championship
100 mm	♀	Best	2023	10.71	10.68	10.66	10.65
		Average		11.06	11.04	11.03	11.03

100 mm	♂	Best	2023	9.80	9.81	9.82	9.83
		Average		10.49	10.47	10.44	10.44

TSPM-time series prediction model; RM – regression model; RBF – artificial neural networks

The verification of the constructed models over a five-year period showed high conformity (R^2^ and j^2^ values) of empirical data to the initial models, obtaining F-statistic values of 62.61 and 105.77, respectively, for female’s and men’s 100 mm results during the considered period. This confirmed the correctness of the models.

Therefore, it was possible to proceed with the construction of predictive models for the years 2025 and 2027. So, the final stage of the research was to determine the predictions for the best and average results of the 100 mm sprint times for female and men for the years 2025 and 2027 using time series prediction models, regression models (Table 7), and Artificial Neural Network (ANN) models (Table 8).

**TABLE 7:** Predictions of the best and average results of the 100 mm female’s and men’s sprint times for the 2025 and 2027 Athletics World Championships.

Variable	World Championships	TSPM	j^2^	RM	R^2^
**FEMALE**					
	
Best	2025	10.77 ± 0.02	0.07	10.75	0.92
Average		11.11 ± 0.07	0.05	11.09	0.96
	
Best	2027	10.76 ± 0.04	0.12	10.75	0.89
Average		11.10 ± 0.011	0.11	11.09	0.90

**MAN**					
Best	2025	9.75 ± 0.01	0.06	9.74	0.92
Average		10.30 ± 0.11	0.09	10.29	0.91
	
Best	2027	9.74 ± 0.05	0.12	9.73	0.88
Average		10.31 ± 0.01	0.15	10.30	0.89

TSPM-time series prediction model; RM – regression model; φ^2^ – convergence coefficient; R^2 -coefficient of determination

**TABLE 8:** Predicted results of the 100 mm sprint times for female and men for the 2025 and 2027 Athletics World Championships, determined by models using artificial neural networks (RBF).

Competition	Gender	Predictions for place	Year	Network Structure Quality	Quality (L)	Quality (T)	Quality (V)	Error (L)	Error (T)	Error (V)
100 mm	♀			RBF 1-3-1	0.97	0.95	0.93	0.003	0.001	0.003

		Best	2025			10.74 ± 0.01				
		Average				11.08 ± 0.05							

		Best	2027			10.72 ± 0.01				
		Average				11.07 ± 0.02				

	♂			RBF 1-3-1	0.95	0.96	0.94	0.002	0.001	0.004

		Best	2025			9.74 ± 0.03				
		Average				10.28 ± 0.05				

		Best	2027			9.74 ± 0.05				
		Average				10.24 ± 0.08				

L-learning, T-testing, V-validation

Simultaneously, the constructed neural network models demonstrated high conformity of input data (quality) and low error values across all model groups (i.e., training, testing, and validation). This indicated very good conformity of empirical data and network learning, as well as predictive capabilities (validation error – refer to Table 7).

### DISCUSSION

This study demonstrated: that progress in 100 mm sprint times is primarily driven by the emergence of exceptionally talented individuals, rather than a consistent improvement among all top athletes. This finding contributes new insights to current scientific knowledge by highlighting that while winning times have improved due to these extraordinary talents, the average performance of other semifinalists has remained relatively constant over the past four decades. This suggests that advancements in sprinting are not uniformly distributed across all competitors, which has important implications for training practices and talent development programs. That there have been meaningful progresses in 100 mm sprint times over the 40-year period of the Athletics World Championships for both men and female. However, these improvements are irregular over time and mostly relate to extraordinary talents, while the average results of the remaining semifinalists remain at a similar level over the course of four decades of competitions. This suggests that progress in sprinting, in both sexes, is driven by the emergence of exceptionally talented individuals who set new world records or achieve world-leading times during the main competition of the season. When considering the Athletics World Championships, this pattern can be observed in athletes such as Lewis, who achieved times of 9.93 s and 9.86 s, followed by Greene a few years later with times of 9.86 s, 9.80 s, and 9.82 s. A decade later, the phenomenal Bolt achieved the best 100 mm sprint result ever of 9.58 s, followed by 9.79 s a few years later [35–37]. More recently, champion sprinters such as Coleman and Lyles have recorded times of 9.76 s and 9.83 s, respectively, in the last two editions of the Athletics World Championships. These exceptionally talented athletes have improved the winning times of the main competitions in the 100 mm races, while the average times of the semifinalists in the Athletics World Championships remains relatively constant, falling between 10.40 and 10.50 s over the 40-year period under analysis.

In the case of male sprinters, it is evident that once in a decade or so, an exceptionally talented individual appears who achieves times close to the world record or even surpasses it. On the other hand, the average times of the semifinalists do not seem to improve significantly over the four decades of Athletics World Championships and deviate significantly from the winners’ results. The same holds true in the case of female. Devers, while winning the Athletics World Championships in 1993, achieved a very fast time of 10.82 s, while Jones sprinted even faster (10.70 s) in 1999. Between 2013 and 2022, perhaps the most talented female sprinter ever, Fraser-Pryce won several championships, reaching times of 10.73 s, 10.71 s, 10.76 s, and 10.67 s. The most recent World championship was won by one of the youngest athletes, Sha’Carri Richardson, with a time of 10.65 s. In comparison to men’s results, the average times of female’s semifinalists have improved significantly over the 40-year period, and they do not deviate as much from the results of the winners. The question arises: what separates elite sprinters from the good ones who reach only the semifinals? It is difficult to distinguish the best sprinters from the good ones based on a single factor such as age, height, or strength-power qualities. This is evident from the success of both very tall sprinters (e.g., Jones, Bolt) and rather short sprinters (Fraser-Pryce, Richardson) [37]. It seems that a variety of factors contribute to sprint performance, making it necessary to consider multiple aspects to distinguish between champions and those who only reach the first round or the semifinals of major events.

Some of these traits can be categorized into morphological, physiological, and motor factors, although race or ethnicity may also play a significant role. Body composition, muscle mass distribution, limb length and proportions, as well as foot and ankle structures, are crucial for elite sprinting performance [38]. Elite sprinters typically possess a higher proportion of fast-twitch muscle fibers in the lower limbs, which are essential for generating higher levels of muscle power and achieving higher speeds [39, 40]. These top-level track and field athletes are also characterized by lower percentages of body fat [39]. In addition, elite sprinters commonly have a greater proportion of muscle mass in the gluteus maximus, quadriceps, calves, and hamstrings [39, 40, 41]. These muscles are highly active during sprinting, as evidenced by EMG studies [41, 42]. Optimal limb length also appears to be a significant advantage for sprinting, with longer legs and a shorter torso being key attributes [10, 11]. Additionally, well-developed and structured feet and ankles provide better force transmission and greater stability during landing and take-off [38].

In the same vein, greater anaerobic capacity allows elite sprinters to maintain high speeds without rapid fatigue. For example, when considering metabolic factors, an enhanced ability to buffer and clear lactate also allows for an increased capacity to sustain high-intensity efforts [43]. A more efficient ATP-PCr system provides immediate energy release. Perhaps the most significant factor that distinguishes elite sprinters from good sprinters is their superior neuromuscular coordination, which enables more controlled and natural powerful movements of antagonistic muscle groups across different limbs [40]. Motor factors can also differentiate elite from good sprinters. Optimal running mechanics, including stride length, stride frequency, and ground contact time, as well as efficient use of the arms, result in a superior sprinting technique that is highly efficient [21, 22]. High levels of strength and power, especially in the muscles around the hips, knees, and ankle joints, enhance starting and acceleration abilities [21]. A faster reaction time is crucial for effective block starts. Good flexibility, particularly in the hips and ankles, contribute to a longer stride and improve sprinting efficiency [4]. Additionally, balance and both intra- and inter-muscle coordination allow for optimal sprinting form and reduce the risk of injury. Mental aspects such as focus, concentration, and the ability to achieve higher levels of performance under pressure are equally indispensable traits for elite sprinters [44]. At the elite level, in addition to the aforementioned attributes, access to quality training programs as well as effective recovery strategies – including nutrition, sleep, and physiotherapy – may differentiate the elite from the good or average ones [45].

This study provided a comprehensive analysis of men’s and female’s 100 mm sprints, spanning several decades of sprinting competitions. By meticulously collecting and analyzing data, the study elucidated trends and dynamics in sprinting performance, highlighting the influence of factors such as age on results. The application of advanced statistical techniques, including time series analysis and predictive modeling, enabled the prediction of future outcomes. Overall, the research relied on data obtained from various sources, including the International Association of Athletics Federations (IAAF) website and specialized athletics journals. Rigorous verification processes ensured data accuracy. Focusing on men’s and female’s 100 mm sprint times from 1983 to 2023, the study constructed a comprehensive database integrating results from specific competitions to maintain consistency across analyses. Correlation analyses revealed relationships between average times, age of participants, and championship winners, emphasizing the role of age in athletic performance. Predictive models, such as regression analysis and neural networks, were developed to predict future outcomes. Non-linear dependencies required the utilization of both linear and non-linear models, and rigorous testing confirmed the accuracy of these models. We also included predictions for 100 mm sprint results for female and men for 2025 and 2027. Neural models accurately predicted the results for 2023, enabling the construction of predictive models for subsequent years. Verification over five years showed high consistency of empirical data with initial models, confirming model accuracy. Neural network models demonstrated high consistency and low error values, indicating excellent predictive capabilities.

Nevertheless, what are the benefits of comparing the average times of finalists and semifinalists to those of World sprint champions? Initially, the motivation for analyzing World Championships data was the possibility of modeling improvement in results and allowing for a comparison of sprint performances by a single athlete at different distances (i.e., obtaining an objective measure of results independent of the distance covered). For example, a 1 km race could be used as an indicator for a 5 km race, and the effect of training could be observed in improved performances, not necessarily at the same distances. Such measures have been considered before. In this context, Purdy et al. [46] developed a scoring system based on changes in the running curve, while Riegel [47] considered the percentage of the world record speed as a measure of performance. However, we utilized data collected from World Championship semifinalists and World Champions to propose a suitable comparison. To characterize changes in results at each distance, we considered the ranges of the best times or the differences between the average times. Distances of 100 mm, 200 m, and 400 m have exceptionally large spreads due to clusters of very good results, likely occurring during significant competitions. This leads to the conclusion that results decrease relative to the world record at approximately a constant ratio over time. Therefore, we have concluded that a better approach would be to compare the best and averaged times within the same distance.

The predicted results are consistent with analyses of Mleczko [48] for the year 2025. The results provided by the author for the year 2025 were 10.67 s for female and 9.67 s for men. Comparing these values with the predictions from the current study, it can be observed that they are in line with the predictions of the neural models (female: 10.76 s ± 0.03 s; men: 9.74 s ± 0.03 s). Nowak [49] determined that the achievable limit results (achievable) for the year 2025 will be 8.42 s for men and 10.11 s for female. This respective research did not yield such results (as it used a different analytical method); however, the values provided by Dutch authors have contributed to the conception of this study. By calculating the percentage improvement in results, assuming raw data from 1985 as the baseline and using indices based on a constant basis, the time improvements were determined to be 5.1% for men (9.74 s in 2025) and 8.5% for female (10.76 s in 2025). Therefore, the model and prognostic results indicate that in the near or slightly longer term, the variability dynamics of the best 100 mm race times will not improve for either men or female. However, for men, the predicted annual increase will be 13.5%, while for female it will be 6.6%.

In summary, the increase in variability dynamics toward the limit result for men will be 6.9% greater than for female. In this context, Einmahl and Magnus [50] established a limit result of 9.29 s for men. Considering this value, female are expected to improve their results by 1.4% more than men in the future. These differences arise from the analytical tools used, such as predictive modeling with neural networks, as opposed to the mathematical modeling employed in this study and by other researchers [30, 31]. Verification will only be possible after comparing these predictions with actual variability indices over the next years. Testing the hypothesis of intergroup differences (female vs. men) using one-way analysis of variance confirms this hypothesis. As a result, significant statistical differences based on gender were identified. Tukey’s post-hoc tests further revealed that female significantly differentiate between groups more than men do.

## CONCLUSIONS

Our findings not only advance the understanding of sprinting performance but also have practical implications for coaches, athletes, and sports organizations in developing new and more effective training and competition strategies. Notably, the dynamics of performance changes differ between male and female athletes. While both groups have shown significant improvements over time, male sprinters have experienced more pronounced leaps in performance, often driven by exceptional talents setting new benchmarks. Female sprinters have also improved, but the progression appears to be more gradual and consistent. Understanding these differences is crucial for tailoring training programs and resource allocation to address the unique needs of each group.

Based on current trends, we can estimate the results that may be required to secure medals at upcoming Olympic Games or World Championships. For male athletes, sprint times below 9.80 seconds are increasingly necessary to contend for gold medals, with silver and bronze medalists often finishing under 9.90 seconds. For female athletes, times under 10.80 seconds may be required for gold, with medalists typically finishing under 11.00 seconds. These estimations highlight the escalating standards in elite sprinting and underscore the importance of continuous improvement and innovation in training methodologies.

Moving forward, additional research in this field, integrating new data and refining predictive models, will enhance our ability to understand and predict competitive results more accurately. By applying the novel insights presented herein, practitioners who are directly involved in the sport science community can optimize their training programs, practices, and strategies, thereby enhancing sport-specific performance, not only in sprinting events but also across a wide range of sports disciplines. Finally, we consider that this type of study can open a new avenue for research among sport scientists, especially those interested in examining and establishing the limits of human performance.
